# Cost-effectiveness of DPYD genotyping prior to capecitabine administration for metastatic breast cancer

**DOI:** 10.1007/s10549-026-07948-y

**Published:** 2026-03-28

**Authors:** Tanvi Chiddarwar, Anne Blaes, Karen Kuntz

**Affiliations:** 1 School of Public Health, University of Minnesota, Minneapolis, USA; 2 School of Medicine, University of Minnesota, Minneapolis, USA

**Keywords:** DPYD genotyping, Capecitabine, Metastatic breast cancer, Cost-effectiveness analysis, Pharmacogenomics

## Abstract

**Purpose:**

Patients with a DPYD genetic deficiency who receive capecitabine are at increased risk of severe, potentially fatal toxicities due to impaired drug metabolism. Genetic testing for this deficiency allows for proactive dose adjustments to mitigate these risks. We evaluated the cost-effectiveness of DPYD genotyping prior to capecitabine administration, followed by dose modification for patients with metastatic breast cancer.

**Methods:**

We developed a state-transition model to simulate health outcomes and costs for a cohort of 62-year-old women with metastatic breast cancer from the perspective of the U.S. healthcare payer. Costs and utilities were derived from the literature to calculate quality-adjusted life years (QALYs) and the incremental cost-effectiveness ratio (ICER) for DPYD genotyping compared to no DPYD genotyping. We conducted deterministic and probabilistic sensitivity analyses to identify factors influencing cost-effectiveness.

**Results:**

The genotyping strategy was cost-effective, with a cost of $2,832 yielding 1.16 QALYs, compared to $2,677 and 1.15 QALYs for the no-genotyping strategy. This resulted in an ICER of $12,916/QALY and $10,333 per life-year-gained. In probabilistic sensitivity analysis, the genotyping strategy was cost-effective in 99% of the simulations, using a willingness-to-pay threshold of $100,000/QALY. Results from scenario analyses testing key assumptions also showed that genotyping is cost-effective.

**Conclusion:**

Our findings support the implementation of DPYD genotyping prior to capecitabine initiation in metastatic breast cancer patients. This strategy exemplifies the value of personalized medicine and pharmacogenomics in improving treatment safety and effectiveness. As sequencing technologies advance and become affordable, integration of genotyping into routine oncology care is increasingly feasible.

**Supplementary Information:**

The online version contains supplementary material available at 10.1007/s10549-026-07948-y.

## Introduction

Breast cancer is the most common cancer among women, accounting for approximately 15% of newly diagnosed cancer cases, with 6% of these cases being metastatic at diagnosis [1]. Among the treatment options for metastatic breast cancer, capecitabine stands out as a widely used monotherapy, especially for those who have triple-negative breast cancer. This oral chemotherapy drug is converted into its active form, 5-fluorouracil (5-FU), a cornerstone of cancer treatment due to its ability to effectively inhibit the growth and division of cancer cells [2]. However, the role of the DPYD gene in capecitabine metabolism is critical. The DPYD gene encodes dihydropyrimidine dehydrogenase (DPD), the enzyme responsible for the rate-limiting step in 5-FU metabolism. Genetic variations in DPYD can lead to reduced or absent DPD activity, causing an accumulation of 5-FU in the body and significantly increasing the risk of severe toxicity [3, 4]. This heightened toxicity can manifest as life-threatening conditions, including diarrhea, bone marrow suppression, gastrointestinal toxicity, hand-foot syndrome, and neutropenia, often requiring hospitalization [4, 5].

The body of evidence supporting the use of DPYD genotyping before initiating capecitabine treatment continues to grow. Meta-analyses across various cancer types have demonstrated a significant association between DPYD variant carriers and treatment-related mortality, emphasizing the importance of identifying these mutations to predict and prevent serious side effects [6, 7]. In metastatic breast cancer patients treated with capecitabine, a study on the clinical implementation of pre-treatment DPYD genotyping highlighted its successful integration into routine practice to reduce the risk of severe fluoropyrimidine toxicities [8]. Importantly, research has shown that using pharmacogenomics to guide treatment decisions-such as adjusting treatment intensity based on DPYD genotyping-does not adversely affect treatment efficacy in variant carriers [9, 10]. Furthermore, the cost of managing toxicities in patients with DPYD variants far exceeds the cost of genetic testing, as these patients are more likely to require hospitalization [9]. Prior cost-effectiveness analyses in colorectal cancer have demonstrated that DPYD genotyping before treatment is a cost-effective strategy [11–13].

Despite growing evidence supporting the clinical and economic benefits of pre-treatment DPYD genotyping, adoption in the United States has historically been more cautious than in Europe. The Clinical Pharmacogenetics Implementation Consortium, which develops evidence-based guidelines for integrating pharmacogenetic information into clinical practice, has recommended genotype-guided dose reductions for patients with decreased fluoropyrimidine metabolism since 2017 [14]. The European Medicines Agency has recommended testing for DPD deficiency before starting fluoropyrimidine-based chemotherapy citing its potential to significantly reduce treatment-related toxicities [15]. Building on this momentum, regulatory guidance in the United States has evolved: in February 2026 the US Food and Drug Administration updated safety labeling for capecitabine and fluorouracil to emphasize the risk of severe toxicity in patients with DPD deficiency and to recommend DPYD testing prior to treatment initiation unless immediate therapy is required [16]. This shift is also reflected in professional discourse with an ASCO Clinical Notice discussing the importance of pretreatment DPYD genotyping in fluoropyrimidine therapy [17]. Together, these developments indicate growing recognition of the role of pharmacogenomic testing in improving the safety of fluoropyrimidine treatment.

Pharmacogenomics offers an opportunity to proactively prevent avoidable toxicities and treatment-related mortality. This study focuses on capecitabine therapy for metastatic breast cancer, a commonly used treatment option for patients who may not respond to or are not candidates for targeted therapies. Although the treatment landscape for metastatic breast cancer has expanded with the introduction of targeted agents and immunotherapy, capecitabine monotherapy remains clinically relevant, particularly for patients with triple-negative breast cancer and those receiving later-line therapy. Our aim was to evaluate the cost-effectiveness of DPYD genotyping prior to capecitabine initiation to guide dose adjustments in metastatic breast cancer patients. The findings from our analysis can provide valuable insights for decision-makers considering the adoption of genotyping as a standard practice in the United States.

## Model

### Model overview

**Fig. 1:**
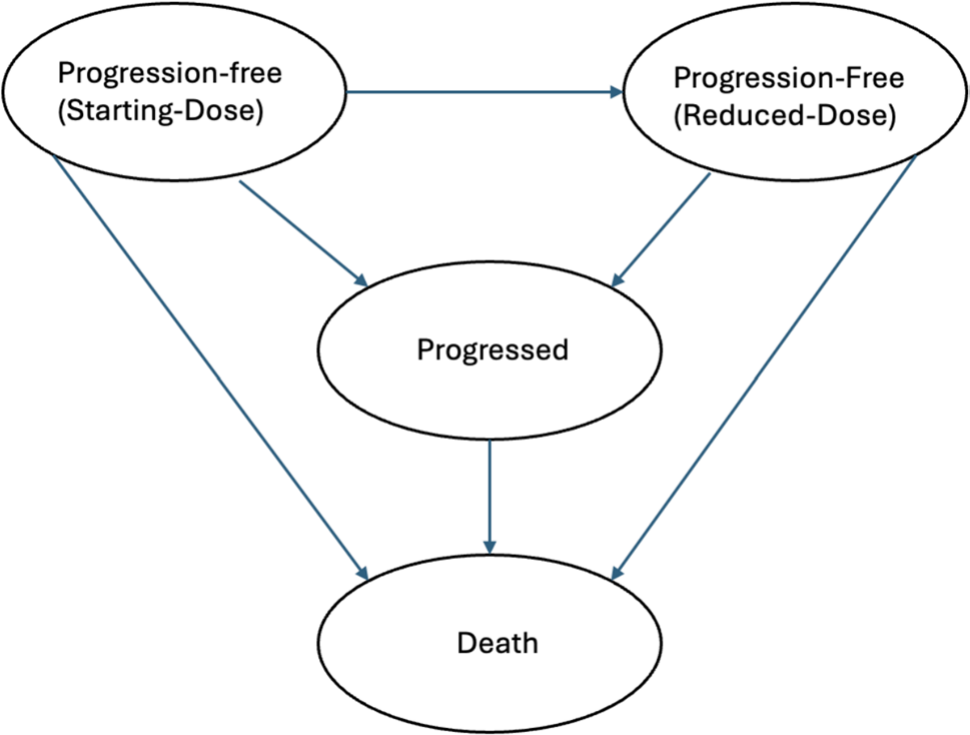
Health-states. The model starts with a hypothetical cohort of patients with metastatic breast cancer, all initially in the progression-free (starting dose) state. From there, patients can either remain in the same state, have a dose reduction, experience disease progression, or die

We developed a cohort state-transition model to evaluate the cost-effectiveness of genotyping prior to administering capecitabine compared to no genotyping for a hypothetical cohort of 62-year-old women newly diagnosed with metastatic breast cancer [18]. The model assessed lifetime costs and quality-adjusted life years (QALYs) from the perspective of the U.S. healthcare payer. It included four health states: Progression-Free (Starting - Dose), Progression-Free (Reduced - Dose), Progressed, and Death, as depicted in Fig. 1.

We built a cohort-state transition model with a monthly cycle length. Costs and QALYs were discounted at an annual rate of 3%, following recommendations from the Second Panel on Cost-Effectiveness in Health and Medicine [19]. These values were used to calculate the incremental cost effectiveness ratio (ICER) of genotyping followed by dose adjustments compared to no genotyping while using a commonly accepted willingness-to-pay threshold of $100,000/QALY [20]. All analyses were conducted using TreeAge Pro 2023 (TreeAge Software Inc., Williamstown, MA, USA), and results were reported in compliance with the Consolidated Health Economic Evaluation Reporting Standards (CHEERS) guidelines, detailed in eTable 1 of the supplement [21].

### Clinical parameters

**Fig. 2:**
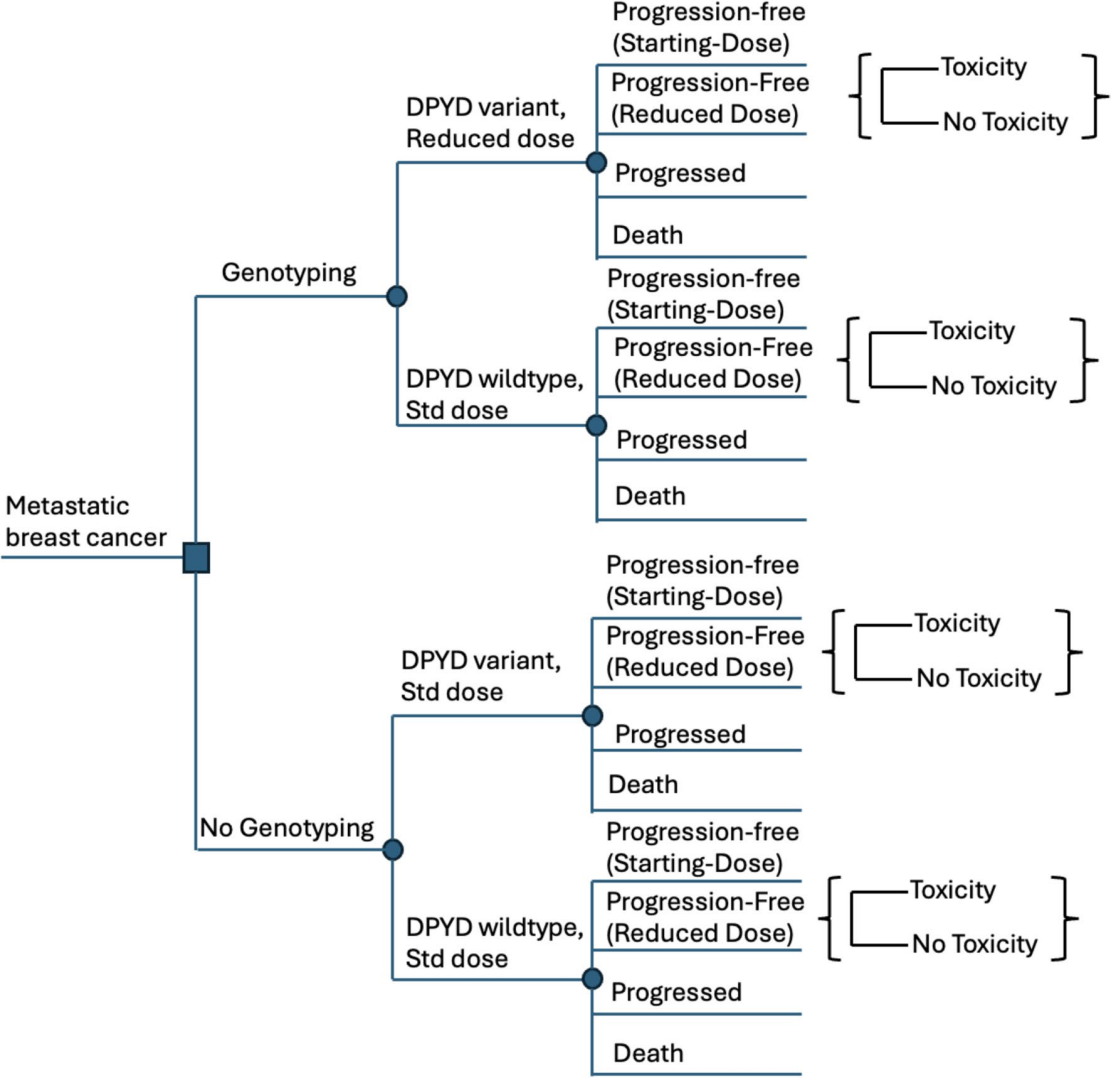
Simplified model framework. In the model, patients either underwent DPYD testing or did not, followed by capecitabine treatment. The diagram illustrates the sequence of events following diagnosis

In our simulated cohort, patients with advanced breast cancer started in the Progression-Free (Starting-Dose) state. If they experienced toxicity, they could transition to the Progression-Free (Reduced-Dose) state. Patients in both Progression-Free states–Starting-Dose and Reduced-Dose–could either progress or die. In the genotyping arm, patients were stratified by their DPYD genotype. People are typically classified as normal, intermediate, or poor metabolizers, with the latter two groups combined as DPYD variant carriers. Those with a DPYD variant received a reduced dose (75%) of capecitabine, while patients with the wild type received the standard dose [14]. Conversely, in the no-genotyping strategy, all patients received the standard dose regardless of genotype. Patients who experienced a toxicity event had their dose reduced to 75% in subsequent cycles and further reduced to 50% if they were already on a reduced-dose regimen. Once a patient progressed, treatment was discontinued as it was deemed ineffective at that stage. Death could result from disease progression, background mortality, or early-treatment-related toxicity; however, we assumed that metastatic breast cancer-related deaths occurred solely due to disease progression. A simplified framework for the model is presented in Fig. 2.

We assumed that dose reduction due to toxicity did not diminish drug effectiveness, as supported by evidence of this phenomenon in metastatic colon cancer [22]. For patients with reduced doses (75%), we also assumed a proportional reduction in both the probability of toxicity and the cost of the drug. Toxicity was defined as grade 3 or 4 chemotherapy-related events, and patients experiencing toxicities were assigned a disutility. Grade 3 toxicities were classified as severe or medically significant but not life-threatening, while grade 4 toxicities were considered life-threatening [23]. Hospitalizations due to toxicity lasted five days and incurred additional costs and disutility.

Our model incorporated parameter estimates from various sources, listed in Table 1. Prevalence of DPYD variants was drawn from existing literature, indicating a range of 3%–8%. Among individuals of European descent, the prevalence was 3%–5%, while it was higher at approximately 8% in individuals of African descent [4, 11, 24]. The probability of disease progression and dying subsequently were calibrated using the progression-free survival and overall survival curves from a randomized clinical trial evaluating capecitabine as a first-line chemotherapy for breast cancer [25]. Background mortality rates were sourced from the CDC US life tables [26].

**Table 1:** Model parameters

Parameter name	Base case	Distributions for probabilistic sensitivity analysis	Source
General information			
Age	62	–	Xiao et al. [37]
Annual discount rate	3%	–	2nd Panel [19]
Body surface area	1.8 m2	–	
Background mortality		–	CDC lifetables [26]
Clinical parameters			
Probability of carrying a DPYD gene variant	0.063	Beta (6.3, 93)	Brooks et al. [11] Dean et al. [4]
Monthly probability of grade 3 or 4 toxicity			Henricks et al. [27]
DPYD variant, standard dose	0.08	Beta (8.6, 94.5)	
DPYD variant, reduced-dose	0.044	Beta (3, 65.5)	
DPYD wild type, standard dose	0.026	Beta (23, 879)	
Monthly probability of hospitalization			Henricks et al. [27]
DPYD variant, standard dose	0.4	Beta (12, 18.9)	
DPYD variant, reduced-dose	0.54	Beta (41.6, 35.4)	
DPYD wild type, standard dose	0.6	Beta (28.4, 18.9)	
Monthly probability of treatment-related death			
DPYD variant, standard dose	0.002	Triangular (0.001, 0.003, 0.002)	Sharma et al. [7]
DPYD variant, reduced-dose	0.0002	Triangular (0.0001, 0.0003, 0.0002)	Brooks et al. [11]
DPYD wild type, standard dose	0.0001	Triangular (0, 0.0002, 0.0001)	Sharma et al. [7]
Monthly transition probabilities			Stockler et al. [25]
Progression-free → Progressed	0.11	Beta (11, 89)	
Progressed → Death	0.051	Beta (5.1, 95)	
Costs ($)			
Cost of capecitabine [HCPCS:J8521]	$0.64/500 mg	-	CMS ASP 2024 [29]
Monthly cost of capecitabine	115.56	Gamma (116, 29)	CMS ASP 2024 [29]
Cost of DPYD genotyping test [CPT: 81232]	174.81	Gamma (174, 44)	CMS Clinical lab fee schedule [28]
Hospitalization cost	12,907	Gamma (12,907, 6450)	Hassett et al. [31]
Health state utilities			
Progression-free	0.715	Beta (7.2, 2.9)	Lloyd et al. [32]
Progressed	0.443	Beta (4.4, 5.6)	Lloyd et al. [32]
Hospitalization (disutility) applied for one week	− 0.28	Beta (28,72)	Brooks et al. [11]
Grade 3/4 toxicity	− 0.125	Beta (13.9, 97)	Sherrill et al. [33]

We calculated the monthly probabilities of experiencing grade 3 or 4 toxicities and hospitalizations by calibrating to a prospective DPYD genotyping study involving patients initiating fluoropyrimidine-based therapy. That study provided separate estimates for individuals with DPYD variants receiving reduced or standard doses and those with wild-type DPYD receiving standard doses [27]. Finally, probabilities of early treatment-related death were derived from studies conducted by Sharma et al. and Brooks et al. [7, 11]

### Costs

We accounted for the costs of DPYD genotyping, capecitabine, and hospitalization for toxicities, as detailed in Table 1, with all costs reported in 2024 U.S. dollars. The cost of DPYD genotyping came from the Clinical Laboratory Fee Schedule of the Centers for Medicare and Medicaid Services (CMS) using the HCPCS code 81232 [28]. Capecitabine costs for a monthly treatment cycle were derived from the CMS Part B Average Sales Price file (June 2024) using the HCPCS code J8521 [29]. Dosing calculations were based on a body surface area of 1.8 m^2^, following the FDA-recommended intermittent regimen of 1,250 mg/m^2^ taken twice daily for 14 days of a 21-day cycle, with treatment continuing until disease progression [30]. Since capecitabine is administered orally, infusion-related costs were not included.

Hospitalization costs associated with adverse events were estimated using data from a real-world study [31]. Costs related to disease management or death were assumed to be equivalent across both arms of the analysis.

### Utilities

Utilities for the progression-free and progressed states of metastatic breast cancer were obtained from a study that used the standard gamble technique to elicit utilities from a general population [32]. Utilities for grade 3/4 toxicities were sourced from a study that employed EQ-5D scores to assess the quality of life in metastatic breast cancer patients treated with capecitabine [33]. The utility associated with hospitalization due to toxicity was estimated in a separate study [11].

### Sensitivity analysis

To assess the impact of model parameters, we performed both deterministic and probabilistic sensitivity analyses. Parameter ranges were sourced from the literature and when unavailable, values were varied by ± 25%. For the probabilistic sensitivity analysis, parameters were varied using prespecified distributions in 10,000 Monte Carlo simulations, with results presented as an ICER scatterplot. We performed one-way sensitivity analyses, with results presented in a tornado diagram, and separately also assessed the effect of population-level DPYD variant probabilities.

## Results

### Base case results

The genotyping strategy was found to be more effective than the no-genotyping strategy. Base-case results from our analysis are presented in Table 2. The estimated life expectancy was 2.23 years for the genotyping arm compared to 2.22 years for the no-genotyping arm. When incorporating quality of life, patients in the genotyping arm had 1.16 QALYs, compared to 1.15 QALYs in the no-genotyping arm. While the genotyping arm was $155 more costly, with total costs of $2,832 versus $2,677 for the no-genotyping arm, it was cost-effective. The analysis resulted in an ICER of $12,916 per QALY and $10,333 per life year gained, which is well below the commonly accepted willingness-to-pay threshold of $100,000/QALY in the United States.

**Table 2:** Results of cost-effectiveness analysis

Strategy	No genotyping	Genotyping
Total cost	$2,677	$2,832
Incremental total cost	–	$155
QALYs	1.15	1.162
Incremental QALY	–	0.012
Life-years	2.219	2.234
Incremental life-years	–	0.015
ICER		
$/QALY	–	12,916
$/Life-year	–	10,333

### Sensitivity/scenario analysis

**Fig. 3:**
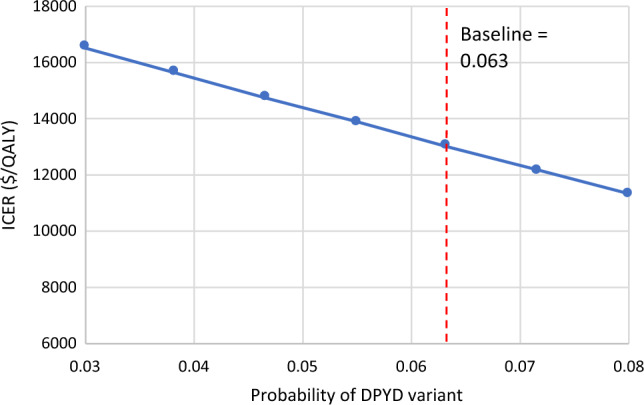
One-way sensitivity analysis–probability of DPYD variant. One-way sensitivity analysis evaluating the impact of varying the probability of a *DPYD* variant on the ICER, with a baseline value of 6.3%. The genotyping strategy remained cost-effective across all tested values at a willingness-to-pay threshold of $100,000/QALY

Results from the deterministic sensitivity analysis are presented in a tornado diagram (eFigure 1), displaying the six most influential parameters, all of which remained well below the $100,000/QALY threshold. We also conducted a one-way sensitivity analysis to examine the impact of varying the population probability of the DPYD variant. While the average probability is 6.3%, we tested a range between 3 and 8%, and the ICER remained below the $100,000/QALY threshold across the entire range, as shown in Fig. 3.

**Fig. 4:**
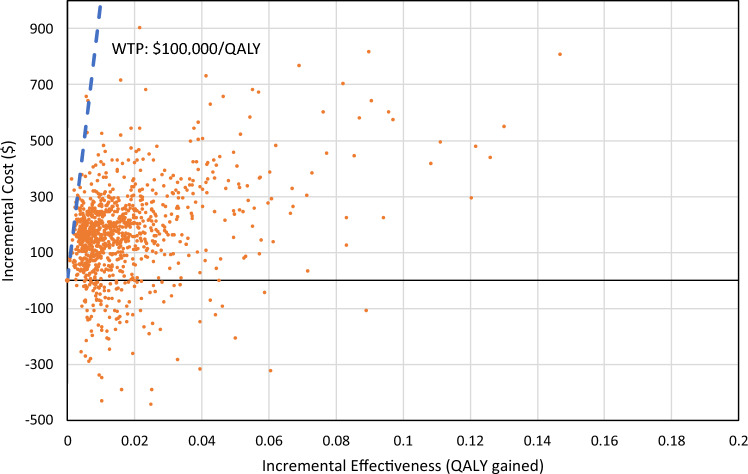
ICER scatterplot. The probabilistic sensitivity analysis, based on 10,000 Monte Carlo simulations, showed that DPYD genotyping was cost-effective in 99% of cases at a willingness-to-pay threshold of $100,000, as illustrated by the ICER scatterplot

For the probabilistic sensitivity analysis, we conducted 10,000 Monte Carlo simulations using prespecified parameter distributions. The results, illustrated in the ICER scatterplot (Fig. 4), indicate that the genotyping strategy is cost-effective in 99% of simulations, including 12% of the simulations where it is cost-saving. In these simulations, the cost of genotyping is fully offset by savings from avoided toxicities, resulting in improved outcomes at a lower overall cost.

## Discussion

To our knowledge, this is the first study to evaluate the cost-effectiveness of DPYD genotyping prior to capecitabine administration compared to no genotyping in metastatic breast cancer patients in the United States. We found that the genotyping strategy was both more effective and slightly more costly, with an ICER of $12,916/QALY, making it cost-effective at the commonly accepted US threshold of $100,000/QALY from the healthcare payer perspective. These findings remained robust across sensitivity analyses. Our results align with findings from a similar study conducted in the UK, the Netherlands, and Hungary, where DPYD testing prior to fluorouracil administration was also shown to be cost-effective [34]. As one of the oldest and widespread chemotherapies, ensuring the safety of fluoropyrimidines should remain a priority. Our study adds to the growing body of evidence supporting the integration of genotyping into routine clinical practice to improve patient outcomes.

Establishing DPYD genotyping as a standard practice in oncology is essential, particularly given that the US National Institutes of Health estimates approximately 1,300 annual deaths attributable to DPD deficiency-equivalent to 0.5% of patients treated with fluoropyrimidines [24]. While the overall probability of harboring DPYD variants is not exceedingly high, the associated healthcare costs and patient disutilities are substantial. Notably, African American individuals carry a disproportionate burden due to the higher prevalence of these variants, highlighting the urgency for widespread testing. A deeper analysis by race and gender further revealed that African American women exhibited the lowest DPD enzyme activity among all race-gender groups, suggesting that women of African descent may face an elevated risk of fluoropyrimidine-related toxicity [35].

Although the FDA has issued safety warnings emphasizing the elevated toxicity risk in individuals with DPD deficiency, translating this information into clinical practice remains a necessity. We urgently need increased awareness and better direction through professional society guidelines. A survey of oncologists revealed a clear gap between awareness and practice: while 98% agreed that patients with DPD deficiency are at higher risk of toxicity, and 96% indicated they would adjust fluoropyrimidine dosing for known deficiency, only 32% considered pretreatment DPYD testing useful for guiding treatment, and just 20% had ever ordered the test. The primary barriers to testing were the perceived low prevalence of DPD deficiency (54%) and the lack of strong clinical practice guideline recommendations (48%) [36]. This disconnect highlights the urgent need for targeted educational initiatives and clearer clinical guidelines to facilitate the integration of DPYD genotyping into standard oncology practice.

We find ourselves in a moment where the tools of genetics allow us to tailor medications to specific populations. Through pharmacogenomics, we can potentially prevent unnecessary suffering and loss of life. Some of the hesitation may come from the lack of prospective studies and trials supporting using genetics and the fear that reducing the dosage may reduce the effectiveness of the drugs. While critics of genetic testing often highlight cost as a barrier, advancements in technology have made genotyping faster and more affordable, with results typically available in under a week. Given the relatively low cost and the ability to prevent severe toxicities, integrating DPYD genotyping into routine oncology chemotherapy practices is increasingly practical and advantageous. Additionally with the advent of Next generation sequencing and increasing amount of people using it, additional DPYD genotyping may not prove to be an additional burden of genotyping.

Our analysis has some limitations that should be considered when interpreting the results. First, we faced challenges in obtaining all necessary parameters, which required us to make certain assumptions. For example, we assumed that reducing the dosage by a specific percentage would proportionally decrease both the risk of toxicity and the associated drug costs. Similarly, we applied disutility only during hospitalization, although severe toxicity may lead to longer-term reductions in quality of life, potentially underestimating the benefit of DPYD genotyping. Second, we did not account for cases of complete DPD deficiency, which may further underestimate the importance of DPYD genotyping, as these patients are at particularly high risk of severe toxicities. Third, our analysis did not incorporate the sensitivity and specificity of the genotyping test. Finally, although capecitabine monotherapy may represent a smaller proportion of metastatic breast cancer treatments as targeted therapies and immunotherapy expand, it remains clinically relevant for specific populations, including patients with triple-negative disease and those receiving later-line therapy. Survival and utility inputs in our model were informed by earlier clinical trials of capecitabine, which may not fully reflect contemporary treatment sequencing in metastatic breast cancer. Future research in this area should focus on conducting more prospective studies to strengthen the evidence supporting routine DPYD genotyping prior to administering fluoropyrimidines.

## Conclusion

Patients with a DPYD variant face an increased risk of toxicities from fluorouracil treatments. In our study, we found that implementing DPYD genotyping prior to initiating capecitabine treatment in metastatic breast cancer patients was cost-effective in the United States. Our findings offer valuable insights for decision-makers evaluating the adoption of genotyping as a standard practice in oncology.

## Supplementary Information

Below is the link to the electronic supplementary material.

Supplementary file1 (DOCX 84 KB)

## Data Availability

No datasets were generated or analysed during the current study.
